# Cul4a attenuates LPS-induced acute kidney injury via blocking NF-kB signaling pathway in sepsis

**DOI:** 10.5937/jomb0-33096

**Published:** 2022-02-02

**Authors:** Jing Zhao, Qiuxia Duan, Cuihong Dong, Jing Cui

**Affiliations:** 1 Yantaishan Hospital, Department of Critical Care Medicine, Yantai, China; 2 The Third People's Hospital of Qingdao, Department of Critical Care Medicine, Qingdao, China; 3 Shandong College of Traditional Chinese Medicine, Yantai, China; 4 The Third People's Hospital of Qingdao, Department of Emergency, Qingdao, China

**Keywords:** sepsis, AKI, Cul4a, oxidative stress, inflammation, apoptosis, sepsa, AKI, Cul4a, oksidativni stres, zapaljenje, apoptoza

## Abstract

**Background:**

Acute kidney injury (AKI) is a common disease that can develop into end-stage kidney disease. Sepsis is one of the main causes of AKI. Currently, there is no satisfactory way to treat septic AKI. Therefore, we have shown the protective function of Cul4a in septic AKI and its molecular mechanism.

**Methods:**

The cellular and animal models of septic AKI were established by using lipopolysaccharide (LPS). Western blot (WB) was employed to analyze Cul4a expression. RT-qPCR was employed to test the expression of Cul4a, SOD1, SOD2, GPX1, CAT, IL-6, TNF-a, Bcl-2, IL1b, Bax and KIM-1 mRNA. ELISA was performed to detect the contents of inflammatory factors and LDH. CCK-8 was utilized to detect cell viability. Flow cytometry was utilized to analyze the apoptosis. DHE-ROS kit was used to detect the content of ROS.

**Results:**

Cul4a was down-regulated in cellular and animal models of septic AKI. Oxidative stress is obviously induced by LPS, as well as apoptosis and inflammation. However, these can be significantly inhibited by up-regulating Cul4a. Moreover, LPS induced the activation of the NF-kB pathway, which could also be inhibited by overexpression of Cul4a.

**Conclusions:**

Cul4awas found to be a protective factor in septic AKI, which could inhibit LPS-induced oxidative stress, apoptosis and inflammation of HK-2 cells by inhibiting the NF-kB pathway.

## Abstract (sr)

**Uvod:**

Akutna insuficijencija bubrega (AKI) je česta bolest koja može prerasti u kritičnu fazu oštećenja bubrega. Sepsa je jedan od glavnih uzroka AKI. Trenutno ne postoji zadovoljavajući način lečenja septičke akutne insficijencije bubrega. Stoga smo pokazali zaštitnu funkciju Cul4a kod septičke insuficijencije bubrega i njegov molekularni mehanizam.

**Metode:**

Ćelijski i životinjski modeli septičke akutne insuficijencije bubrega su izazvani upotrebom lipopolisaharida (LPS). Za analizu ekspresije Cul4a korišćen je Vestern blot (WB). RT-qPCR je korišćen za ispitivanje ekspresije mRNA Cul4a, SOD1, SOD2, GPKS1, CAT, IL-6, TNF-a, Bcl-2, IL1b, Bax-a i KIM-1 mRNA. Izvršena je ELISA analiza za otkrivanje sadržaja upalnih faktora i LDH. Za utvrđivanje održivosti ćelija korišćen je CCK-8. Za analizu apoptoze korišćena je protočna citometrija. Za otkrivanje sadržaja ROS-a korišćen je DHE-ROS komplet.

**Rezultati:**

Cul4a je snižen u ćelijskim i životinjskim modelima septičkog AKI. Oksidativni stres je očigledno izazivan LPS-om, kao i apoptoza i upala. Međutim, oni se mogu značajno inhibirati regulacijom Cul4a. Štaviše, LPS je indukovao aktivaciju NF-kB signalnog puta, što bi takođe moglo biti inhibirano prekomernom ekspresijom Cul4a.

**Zaključak:**

Utvrđeno je da je Cul4a zaštitni faktor u slučajevima akutne septičke insuficijencije bubrega, što, inhibirajući NF-kB signalni put, može sprečiti oksidativni stres izazvan LPS-om, apoptozu i upalu ćelija HK-2.

## Introduction

AKI refers to a syndrome in which a patient's renal function is significantly declined rapidly due to various causes, and a series of clinical symptoms occur, including increased serum creatinine (Cr), decreased urine output, electrolyte disturbance, acidbase imbalance, etc. In severe cases, acute brain edema, acute heart failure, or even life-threatening may occur. Suffering from AKI can lead to increased demand for renal replacement therapy, increased risk of death, and more treatment costs, which will bring a heavy burden to patients and society [1] [2] [3]. AKI could be induced by many factors, including sepsis, ischemia-reperfusion injury, and nephrotoxic drugs. A large clinical study showed that in critically ill patients, septic shock is the main factor in the onset of AKI, accounting for 47.5% of the total population [4].

Sepsis is a continuous and excessive inflammatory response and immunosuppression caused by pathogen invasion. It is an important factor for organ failure of the body and can lead to shock and even death of patients [5]. Sepsis is a common clinical systemic critical illness. In critically ill patients, the fatality rate reaches 35% [6], which seriously threatens the life and health of patients [7]. Studies found that sepsis patients had a 50% risk of developing AKI, and such patients have a poor prognosis and high mortality [8] [9]. In clinical practice, the treatment of septic AKI is often implemented through strategies such as fluid replacement, diuretics and antibiotics. However, the fatality rate of septic AKI has not been significantly reduced [10]. Therefore, exploring new sepsis AKI treatment drugs is of great significance for alleviating kidney damage and saving patients' lives and health.

Cullin4A (Cul4a) belongs to the E3 ligase ubiquitin family in the ubiquitin-proteasome system (UPS) and determines the substrate specificity of ubiquitination modification [11] [12] [13]. Cul4a ubiquitin ligase has a wide range of substrates and acts a pivotal part in a series of biological processes such as signal transduction, transcription regulation, cell cycle regulation, maintenance of genome stability, and embryo development [14] [15]. At present, Cul4a has been extensively studied in various cancers [16] [17], but its function in kidney disease such as septic AKI is still unclear.

Here, we describe a new role of Cul4a, which is to inhibit inflammation and apoptosis mediated by oxidative stress in septic AKI. This will provide a potential new treatment for septic AKI.

## Materials and Methods

### Rat septic AKI model

Ten male Sprague-Dawley (SD) rats (Shanghai Experimental Animal Center of Chinese Academy of Sciences) were raised in an SPF environment. The breeding room has a temperature of 22-25°C and a humidity of about 50%. LPS (5 mg/kg) was injected intraperitoneally to establish a septic AKI model.

### Cell treatment

HK-2 cells, human renal cortex proximal convoluted tubule epithelial cell line, were purchased from Yaji Biotechnology Co., Ltd (Shanghai, China). The cells were cultured in a culture medium composed of DMEM/F-12 (Gibco, Rockville, MD, USA) and 10% FBS (Gibco, Rockville, USA) at 37°C with 5% CO_2_. The medium needs to be changed every 24 hours. 500 ng/mL LPS was used to induce cell damage.

The Cul4a overexpression plasmids (SangonBiotech, Shanghai, China) was transfected into HK-2cells using Lipofectamine™ 3000 in accordance withthe instructions.

### Western blot

Radioimmunoprecipitation assay lysis buffer (Beyotime, Shanghai, China) was used to extract the total protein in HK-2 cells. The concentration was examined by the BCA method. After incubation with the loading buffer, the same amount of protein (30 mg) from each group was added into SDS-PAGE. The voltage was set to 120 volts. When the protein is sufficiently separated, it is transferred to the PVDF membrane. The current was set to 300 mA. After the membranes were blocked by QuickBlock™ Blocking Buffer (Beyotime, Shanghai, China), primary antibodies (Cul4a, Abcam, Cambridge, MA, USA, Rabbit, 1:1000; IκKα, Abcam, Cambridge, MA, USA, Rabbit, 1:1000; IκBα, Abcam, Cambridge, MA, USA, Rabbit, 1:1000; GAPDH, Abcam, Cambridge, MA, USA, Rabbit, 1:1000) were added and incubated at 4°C. The next day, the secondary antibody was used to incubate the membranes. The electrochemiluminescence (ECL) developer was added dropwise to develop imaging, and the grey value was semi-quantitatively analyzed according to Image J software.

### RT-qPCR analysis

The TRIzol reagent (Invitrogen, Carlsbad, CA, USA) was used to extract total RNA in HK-2 cells following the protocols. The complementary deoxyribose nucleic acid (cDNA)was synthesized using Real Master Mix (Bio-Rad, Hercules, CA, USA). RT-qPCR was performed using the Prism 7900 System. GAPDH was utilized to normalize the expression of mRNAs. All the primers were listed in Table 1.

**Table 1:** Real-time PCR primers RT-PCR, quantitative reverse-transcription polymerase chain reaction

Gene name	Forward (5’>3’)	Reverse (5’>3’)
Cul4a	CAAGACAGGGAGGTTCCA	TCTCCACACAGGCAATCA
TNF-α	AGGCACTCCCCCAAAAGATG	CCACTTGGTGGTTTGTGAGTG
IL-1b	ATGCCACCTTTTGACAGTGATG	GAAGGTCCACGGGAAAGACA
IL-6	GCCTTCTTGGGACTGATGCT	CTGCAAGTGCATCATCGTTGT
SOD1	CAATGTGGCTGCTGGAA	TGATGGAATGCTCTCCTGA
SOD2	GCCGTGTTCTGAGGAGAG	GTCGTAAGGCAGGTCAGG
GPX1	TTGAGAAGTGCGAGGTGAA	TCCGCAGGAAGGTAAAGAG
CAT	TGGTTTTCACCGACGAG	TTTGCCTTGGAGTATCTGG
Bcl-2	GACTGAGTACCTGAACCGGCATC	CTGAGCAGCGTCTTCAGAGACA
Bax	CAGTTGAAGTTGCCATCAGC	CAGTTGAAGTTACCATCAGC
IκKα	AAACCAGAAAATTGTTGTGGACT	ATCGAATCCCAGACCCTATATCAC
IκBα	TAAGCAAAATCCTGACCTGGTGT	GCTCGTCCTCTGTGAACTCC
GAPDH	ACAACTTTGGTATCGTGGAAGG	GCCATCACGCCACAGTTTC

### Determination of malondialdehyde (MDA)

The level of MDA in the supernatant of HK-2 cells was examined by Lipid Peroxidation (MDA) Assay Kit (Abcam, Cambridge, MA, USA) according to the instructions.

### Determination of ROS production

The contents of ROS in HK-2 cells was tested using DCFH-DA (MCE, Nanjing, China). The cells were incubated with DCFH-DA (5 mmol/L) for 30 min in the dark. Then the cells were collected by 0.05% trypsin-EDTA solution. After that, the cells were suspended in a fresh medium. Finally, the level of ROS was tested by a flow cytometer.

### Enzyme-linked immunosorbent assay (ELISA)

The supernatant of HK-2 cells was collected. The contents of inflammatory cytokines (IL-6, IL-1b, TNF-a) and LDH were detected by commercial ELISA kits (Elabscience, Wuhan, China) following the protocols.

### Flow Cytometry

The HK-2 cells were collected by trypsin and centrifugation. Then the cells were resuspended in a 200 mL binding buffer. After that, 5 mL Annexin V-FITc and 5 mL PI were added into the binding buffer. Finally, 10 min later, the apoptosis rate was measured by a flow cytometer.

### TUNEL staining

The apoptosis was examined by TUNEL staining with a TUNEL kit (Roche, USA) following the manufacturers' instructions. The nucleus was stained by DAPI. The images were observed by the inverted fluorescence microscope.

### Statistical analysis

The measurement data were described as the mean±standard deviation (SD). All statistical analyses were performed by GraphPad Prism 8.0. One-way analysis of variance (ANOVA) or Student's t-test was used for comparison. Significance was accepted at P < 0.05.

## Results

### Cul4a was down-regulated in LPS-treated HK-2 cells

**Figure 1:**
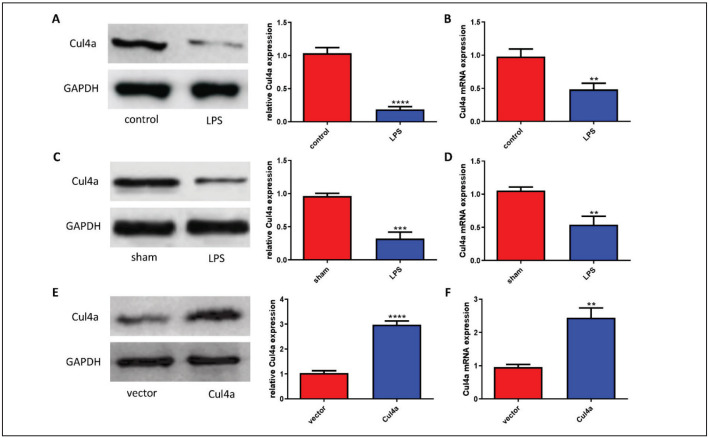
Cul4a was down-regulated in LPS-treated HK-2 cells (A) Western blot showed the expression of Cul4a in HK-2 cells(“****” p < 0.0001 vs. control, n = 3)<br<(B) Cul4a mRNA expression in HK-2 cells was detected by RT-PCR (“**” p < 0.01 vs. control,n = 3)<br>(C) Western blot showed the expression of Cul4a in kidney tissues of rats (“***” p < 0.001 vs. sham, n = 3)<br>(D) Cul4a mRNAexpression in kidney tissues of rats was detected by RT-PCR (“**” p < 0.01 vs. sham, n = 3)<br>(E) The expression of Cul4a in HK-2cells transfected with plasmids (“***” p < 0.001 vs. control, n = 3)<br>(F) Cul4a mRNA expression in HK-2 cells transfected with plasmids was detected (“**” p < 0.01 vs. control, n = 3)

First, the expression of Cul4a in the LPS-treated HK-2 cells was detected through WB. Compared with the control group, Cul4a expression in HK-2 cells of the LPS group was significantly reduced (Figure 1A). At the same time, the level of Cul4a mRNA was also examined, and the result was consistent with the protein level (Figure 1B). In addition, we constructed the rat model of septic AKI and also detected Cul4a expression in the kidney. Cul4a expression in the LPS group was less than that in the sham group (Figure 1C and Figure 1D). To further study the function of Cul4a, we transfected the Cul4a overexpression plasmid into cells and verified the transfection efficiency of the plasmid from the protein and mRNA levels (Figure 1E and Figure 1F).

### Overexpression of Cul4a inhibited LPS-induced oxidative stress

**Figure 2:**
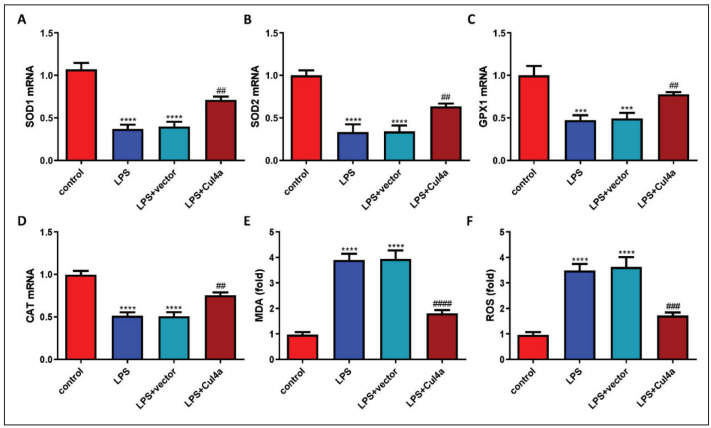
Overexpression of Cul4a inhibited LPS-induced oxidative stress of HK-2 cells (A~D) The levels of SOD1, SOD2, GPX1,CAT mRNA were detected through RT-qPCR(“***” p < 0.001 vs. control, “****” p < 0.0001 vs. control, “##” p < 0.01 vs. LPS+vector, n = 3)<br>(E) The contents of MDA were detected (“****” p < 0.0001 vs. control, “####” p < 0.0001 vs. LPS+vector, n = 3)<br>(F)The production of ROS in HK-2 cells was detected (“****” p < 0.0001 vs. control, “###” p < 0.001 vs. LPS+vector, n = 3)

Through RT-qPCR, we detected the levels of SOD1, SOD2, GPX1, and CAT mRNA. Compared with the control group, the levels of those mRNA in the LPS group were remarkably reduced, suggesting that oxidative stress exists in septic AKI. However, compared with the LPS+vector group, the levels of those mRNA in the LPS+Cul4a group were significantly increased ( Figure 2A, Figure 2B, Figure 2C and Figure 2D). The content of MDA in the cell supernatant was also detected. Overexpression of Cu14a notably inhibited the content of MDA induced by LPS (Figure 2E). In addition, Cul4a also markedly decreased the production of ROS induced by LPS (Figure 2F).

### Overexpression of Cul4a inhibited LPS-induced inflammation

**Figure 3:**
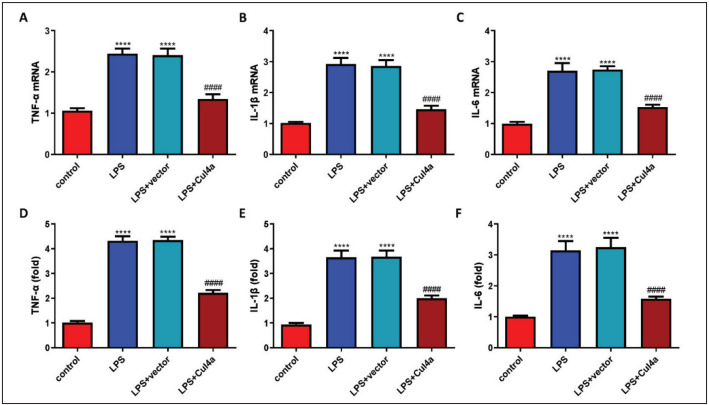
Overexpression of Cul4a inhibited LPS-induced inflammation of HK-2 cells (A~C) The levels of TNF-a, IL-1b, IL-6 mRNAwere detected through RT-qPCR (“****” p<0.0001 vs. control, “####” p<0.0001 vs. LPS+vector, n=3)<br>(D~F) The contents ofTNF-a, IL-1b, IL-6 in the supernatant were detected (“****” p<0.0001 vs. control, “####” p<0.0001 vs. LPS+vector, n=3)

We also tested the inflammatory response in HK-2 cells. LPS obviously induced the production of inflammatory cytokines (IL-6, IL-1b, TNF-α) mRNA in HK-2 cells. While up-regulating Cul4a suppressed their expression (Figure 3A, Figure 3B and Figure 3C). We also tested the contents of inflammatory cytokines in the cell super-natant. The content of inflammatory cytokines in the LPS+Cul4a group was markedly lower than those in the LPS+vector group (Figure 3D, Figure 3E and Figure 3F).

### Overexpression of Cul4a inhibited LPS-induced apoptosis

**Figure 4:**
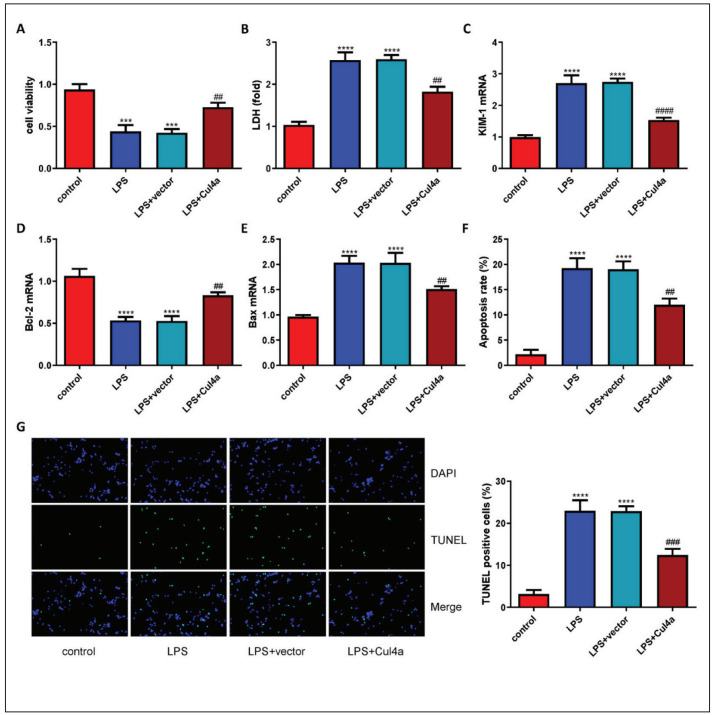
Overexpression of Cul4a inhibited LPS-induced apoptosis of HK-2 cells (A) The viability of HK-2 cells was detected (“***”p < 0.001 vs. control, “##” p < 0.01 vs. LPS+vector, n = 3)<br<(B) The contents of LDH in the supernatant were detected (“****”p < 0.0001 vs. control, “##” p < 0.01 vs. LPS+vector, n = 3)<br>(CDE) The levels of KIM-1, Bcl-2, Bax mRNA were detected (“****”p < 0.0001 vs. control, “##” p < 0.01 vs. LPS+vector, “####” p < 0.0001 vs. LPS+vector, n = 3)<br>(F) The rate of apoptosis wasdetected by flow cytometry (“****” p < 0.0001 vs. control, “##” p < 0.01 vs. LPS+vector, n = 3)<br>(G) Results of TUNEL staining ineach group (200×) (“****” p < 0.0001 vs. control, “###” p < 0.001 vs. LPS+vector, n = 3)

Through the CCK-8 assay, the cell viability was detected. The treatment of LPS significantly reduced the viability of HK-2 cells, but overexpression of Cul4a could reverse this (Figure 4A). Furthermore, overexpression of Cu14a can also reduce the release of LDH (Figure 4B). We also tested the expression of KIM-1 mRNA and found that LPS can significantly induce the expression of KIM-1, while Cul4a can reduce this (Figure 4C). The expression of apoptosisrelated genes was also detected. The level of Bcl-2 mRNA in the LPS treatment group was significantly reduced, while the level of Bax was significantly increased. Overexpression of Cu4a can reverse the above results (Figure 4D and Figure 4E). In addition, the rate of apoptosis was tested through flow cytometry and TUNEL staining. The results suggested that overexpression of Cul4a can inhibit LPS-induced apoptosis of HK-2 cells (Figure 4F and Figure 4G).

### Cul4a inhibited the NF-kB pathway

**Figure 5:**
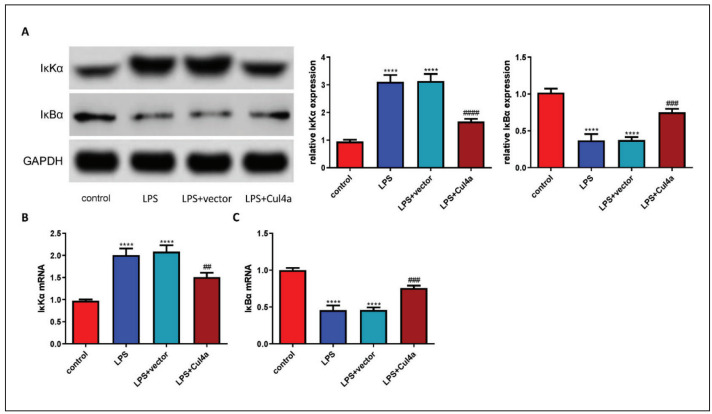
Cul4a inhibited the NF-κB signalling pathway (A) The expression of IκKα and IκBα was detected (“****” p < 0.0001 vs.control, “###” p < 0.001 vs. LPS+vector, “####” p < 0.0001 vs. LPS+vector, n = 3)<br>(B and C) The levels of IκKα and IκBα mRNAwere detected (“****” p < 0.0001 vs. control, “##” p < 0.01 vs. LPS+vector, “###” p < 0.001 vs. LPS+vector, n = 3)

Since the NF-kB pathway plays an important role in oxidative stress, inflammation, and apoptosis, we tested the marker proteins of this signalling pathway. The treatment of LPS significantly increased the expression of IκKα but decreased the expression of IκBα. However, overexpression of Cul4a significantly reversed their expression (Figure 5A). The expression of IκKα mRNA and IκBα mRNA was also detected, and the results were consistent with the previous results (Figure 5B and Figure 5C).

## Discussion

In this present study, we revealed the protective role of Cul4a in septic AKI. We have revealed for the first time that Cul4a is down-regulated in septic AKI. Overexpression of Cul4a can significantly inhibit oxidative stress, inflammation and apoptosis, thereby reducing septic AKI. This protective effect was achieved at least in part by inhibiting the NF-κB signalling pathway.

The pathogenesis of AKI caused by sepsis is very complicated, which may be related to the increase of inflammatory factors, oxidative stress, and apoptosis [18] [19]. Some scholars have suggested that inhibiting apoptosis, improving immune inflammation, and oxidative stress damage can help prevent AKI caused by sepsis [20]. Apoptosis, especially renal tubular epithelial cell apoptosis, plays a key role in septic AKI. Sepsis may cause kidney cell apoptosis through endoplasmic reticulum stress, death receptor pathway, and mitochondrial pathway [21]. In addition, sepsis can cause the body's systemic immune-inflammatory response and oxidative stress damage. Inflammation and oxidative stress can damage the glomeruli and renal tubules, leading to kidney damage, and, ultimately, renal insufficiency [22]. Inflammatory factors such as IL-6 and TNF-a and oxidative stress indicators such as SOD can be used as early diagnostic markers for AKI caused by sepsis, and these markers have important values for judging the prognosis of the disease. Many studies have shown that inhibiting inflammatory factors and oxidative stress can help improve septic AKI. Chen et al. [23] proved that hydrogen sulfide could reduce septic AKI by inhibiting inflammation and oxidative stress. Rutin has also been shown to alleviate septic AKI in mice by inhibiting oxidative stress, inflammation, and apoptosis in the kidney [24].

NF-κB is a nuclear transcription factor involved in inflammation, oxidative stress and apoptosis [25] [26]. Studies have found that the expression of NF-κB in the kidney tissue of septic AKI rats increases. When NF-κB translocates to the nucleus, it can activate the downstream inflammatory factors and oxidative stress indicators such as SOD, etc., thereby promoting the aggravation of inflammatory response and oxidative stress damage and aggravating kidney damage. And inhibition of the NF-κB signalling pathway could reduce renal inflammation, oxidative stress, and apoptosis, thereby reducing renal injury.

Our study demonstrates the regulatory role of Cul4a in septic AKI. Overexpression of Cul4a can remarkably inhibit LPS-induced oxidative stress and inhibit the production of inflammatory factors and apoptosis of HK-2 cells. The protective effect of Cul4a is at least partially achieved by inhibiting the NF-κB pathway.

## Conclusion

To sum up, Cul4a was found to be a protective factor in septic AKI, and overexpression of Cul4a could inhibit LPS-induced oxidative stress, inflammation, and apoptosis of HK-2 cells by inhibiting the NF-κB pathway.

## Dodatak

### Conflict of interest statement

The authors declare that they have no conflict ofinterest.

## References

[1] Li P K, Burdmann E A, Mehta R L (2013). Acute kidney injury: Global health alert. Transplantation.

[2] Xu X, Nie S, Liu Z, Chen C, Xu G, Zha Y, Qian J, Liu B, Han S, Xu A, Xu X, Hou F F (2015). Epidemiology and Clinical Correlates of AKI in Chinese Hospitalized Adults. Clin J Am Soc Nephrol.

[3] Malhotra R, Kashani K B, Macedo E, Kim J, Bouchard J, Wynn S, Li G, Ohno-Machado L, Mehta R (2017). A risk prediction score for acute kidney injury in the intensive care unit. Nephrol Dial Transplant.

[4] Uchino S, Kellum J A, Bellomo R, Doig G S, Morimatsu H, Morgera S (2005). Acute renal failure in critically ill patients: A multinational, multicenter study. JAMA.

[5] Cecconi M, Evans L, Levy M, Rhodes A (2018). Sepsis and septic shock. Lancet.

[6] Mayeux P R, Macmillan-Crow L A (2012). Pharmacological targets in the renal peritubular microenvironment: Implications for therapy for sepsis-induced acute kidney injury. Pharmacol Ther.

[7] Yende S, Austin S, Rhodes A, Finfer S, Opal S, Thompson T (2016). Long-Term Quality of Life Among Survivors of Severe Sepsis: Analyses of Two International Trials. Crit Care Med.

[8] Roberts J A, Choi G Y S, Joynt G M, Paul S K, Deans R, Peake S, Cole L, Stephens D, Bellomo R, Turnidge J, Wallis S C, Roberts M S, Roberts D M, Lassig-Smith M, Starr T (2016). SaMpling Antibiotics in Renal Replacement Therapy (SMARRT): An observational pharmacokinetic study in critically ill patients. BMC Infect Dis.

[9] Sood M M, Shafer L A, Ho J, Reslerova M, Martinka G, Keenan S, Dial S, Wood G, Rigatto C, Kumar A (2014). Early reversible acute kidney injury is associated with improved survival in septic shock. J Crit Care.

[10] Gaieski D F, Edwards M J, Kallan M J, Carr B G (2013). Benchmarking the Incidence and Mortality of Severe Sepsis in the United States. Crit Care Med.

[11] Biedermann S, Hellmann H (2011). WD40 and CUL4-based E3 ligases: Lubricating all aspects of life. Trends Plant Sci.

[12] Sarikas A, Hartmann T, Pan Z Q (2011). The cullin protein family. Genome Biology.

[13] Hochstrasser M (2006). Lingering Mysteries of Ubiquitin-Chain Assembly. Cell.

[14] Sharma P, Nag A (2014). CUL4A ubiquitin ligase: A promising drug target for cancer and other human diseases. Open Biol.

[15] Iovine B, Iannella M L, Bevilacqua M A (2011). Damage-specific DNA binding protein 1 (DDB1): A protein with a wide range of functions. Int J Biochem Cell Biol.

[16] Hung M S, Chen Y C, Lin P, Li Y C, Hsu C C, Lung J H, You L, Xu Z, Mao J, Jablons D M, Yang C (2019). Cul4A Modulates Invasion and Metastasis of Lung Cancer through Regulation of ANXA10. Cancers (Basel).

[17] Sui X, Zhou H, Zhu L, Wang D, Fan S, Zhao W (2017). CUL4A promotes proliferation and metastasis of colorectal cancer cells by regulating H3K4 trimethylation in epithelial-mesenchymal transition. Onco Targets Ther.

[18] Al-Harbi N O, Nadeem A, Ahmad S F, Alanazi M M, Aldossari A A, Alasmari F (2019). Amelioration of sepsis-induced acute kidney injury through inhibition of inflammatory cytokines and oxidative stress in dendritic cells and neutrophils respectively in mice: Role of spleen tyrosine kinase signaling. Biochimie.

[19] Al-Harbi N O, Nadeem A, Ahmad S F, Alotaibi M R, Alasmari A F, Alanazi W A (2018). Short chain fatty acid, acetate ameliorates sepsis-induced acute kidney injury by inhibition of NADPH oxidase signaling in T cells. Int Immunopharmacol.

[20] Xia S, Lin H, Liu H, Lu Z, Wang H, Fan S, Li N (2019). Honokiol Attenuates Sepsis-Associated Acute Kidney Injury via the Inhibition of Oxidative Stress and Inflammation. Inflammation.

[21] Koçkara A, Kayataş M (2013). Renal Cell Apoptosis and New Treatment Options in Sepsis-Induced Acute Kidney Injury. Ren Fail.

[22] Mukhopadhyay P, Eid N, Abdelmegeed M A, Sen A (2018). Interplay of Oxidative Stress, Inflammation, and Autophagy: Their Role in Tissue Injury of the Heart, Liver, and Kidney. Oxid Med Cell Longev.

[23] Chen Y, Jin S, Teng X S, Hu Z, Zhang Z, Qiu X, Tian D, Wu Y (2018). Hydrogen Sulfide Attenuates LPS-Induced Acute Kidney Injury by Inhibiting Inflammation and Oxidative Stress. Oxid Med Cell Longev.

[24] Khajevand-Khazaei M R, Mohseni-Moghaddam P, Hosseini M, Gholami L, Baluchnejadmojarad T, Roghani M (2018). Rutin, a quercetin glycoside, alleviates acute endotoxemic kidney injury in C57BL/6 mice via suppression of inflammation and up-regulation of antioxidants and SIRT1. Eur J Pharmacol.

[25] Ansari M A, Raish M, Ahmad A, Alkharfy K M, Ahmad S F, Attia S M, Alsaad A M S, Bakheet S A (2017). Sinapic acid ameliorate cadmium-induced nephrotoxicity: In vivo possible involvement of oxidative stress, apoptosis, and inflammation via NF-cB downregulation. Environ Toxicol Pharmacol.

[26] Yuan H, Du S, Deng Y, Xu X, Zhang Q, Wang M, Wang P, Su Y, Liang X, Sun Y, An Z (2019). Effects of microRNA-208a on inflammation and oxidative stress in ketamine-induced cardiotoxicity through Notch/NF-cB signal pathways by CHD9. Biosci Rep.

